# A new creep model for studying the non-linear viscoelastic behavior of cooked white, brown and germinated brown Thai jasmine rice by large deformation testing

**DOI:** 10.1016/j.heliyon.2018.e00745

**Published:** 2018-08-20

**Authors:** Panmanas Sirisomboon, Jetsada Posom

**Affiliations:** a Department of Agricultural Engineering, Faculty of Engineering, King Mongkut's Institute of Technology Ladkrabang, 10520, Thailand; b Department of Agricultural Engineering, Faculty of Engineering, Khon Kaen University, 40002, Thailand; c Applied Engineering for Important Crops of the North East Research Group, Department of Agricultural Engineering, Faculty of Engineering, Khon Kaen University, 40002, Thailand

**Keywords:** Food analysis

## Abstract

A new creep model with three parameters for non-linear viscoelastic behavior is proposed as ${\text{ε}}_{\text{t}}\;={\;\text{ε}}_{0}\left(1+\;\frac{{\text{t}}^{\text{n}}}{{\text{k}}_{1}\;+{\;\text{k}}_{2}\;{\text{t}}^{\text{n}}}\right)$, where the applied stress is constant, ${\text{ε}}_{\text{t}}$ is the strain at retardation time (t), ${\text{ε}}_{0}$ is the initial strain and ${\text{k}}_{1}$, ${\text{k}}_{2}$ and n are constants. The relationship has been proved using data derived from cooked Thai Jasmine rice including white, brown and germinated brown rice samples. The creep test at high strain was conducted on scoops of cooked rice using a compression test rig. The model developed showed very accurate prediction performance with coefficients of determination (R^2^) between 0.9991–0.9992 and residual standard errors (RSE) between 0.00030–0.0004.

## 1 Introduction

Rice is commonly consumed in a milled form, which is generally referred to as white rice. This is produced by removing the hull and bran layers of the rough rice kernel in dehulling and milling processes, respectively (Simonelli et al., 2017). However, for health reasons, increasing numbers of people are consuming brown rice and germinated brown rice. Germination significantly changes the biochemical, nutritional and sensory characteristics of brown rice resulting in the degradation of its constituent proteins and carbohydrates, as well as promoting the synthesis and accumulation of biofunctional compounds (Cáceres et al., 2017). The germination process increases the levels of vitamins, minerals, fibres and phytochemicals such as ferulic acid, GABA, γ-oryzanol and also antioxidant activity (Cho and Lim, 2016).

The viscoelastic behavior of food can be analyzed by rheological models. A relatively simple test for obtaining a rheological model is the creep test, where a constant stress is applied on the specimen and the strain is observed. The test subjects can be divided into two categories, low strain (usually less than 5%) for linear viscoelastic behavior and high strain (more than 5%) for non-linear viscoelastic behavior. The well known generalized Kelvin-Voigt model can be used to explain this behavior, and has been applied to foodstuffs including melon tissue (Martinez et al., 2005), cooked spaghetti (Sozer et al., 2008) and wheat kernels (Hernandez-Estrada et al., 2012). Nonetheless, food generally exhibits non-linear viscoelastic behavior when subjected to high strain due to the processing methods used, as well as the eating process. Studies elucidating this behavior of food include; wheat flour dough (Bockstaele et al., 2011), roasted turkey breast (Myhan et al., 2012) tofu and gellan gum gels (Truong and Daubert, 2000), alginate gels (Zhang et al., 2007). No studies on cooked white (milled), brown, and germinated brown Thai Jasmine rice have been reported.

The objective of this research was to characterize the viscoelastic behavior of cooked Thai Jasmine white, brown and germinated brown rice. An investigation of the creep behavior of 2 different non-linear viscoelastic modeling methods i.e. Peleg (1980), Purkayastha et al. (1984) have been undertaken here and a new model has also been proposed.

## 2 Materials and methods

### 2.1 Rice samples and cooked rice preparation

White, brown, and germinated brown rice samples of *Oryza sativa* L., Jasmine rice, were purchased from a commercial outlet in Bangkok, Thailand. The samples were prepared based on the previously reported cooking methods by Sirisomboon et al. (2017). The 200 g of each type of rice sample analyzed required the use of different water to rice ratios as recommended by the rice factories producers, i.e., 2.5:1 for brown rice; 1.6:1 for germinated brown rice; and 1:1 for milled rice, were cooked. A total of 9 replicates per sample were undertaken for each rice type. The cooked rice samples were then subjected to the creep test. The moisture content of the rice samples were measured according to a gravimetric method using a hot air oven at 105 °C. Samples were heated until a constant weight was observed. Five replicates were performed for each sample.

### 2.2 Microstructure of the freeze dried cooked rice

The cooked white, brown, and germinated brown rice samples were frozen at −80 °C for 20 h using a dry cube (F570, Eppendorf, UK) freezer before being subjected to a freeze dryer chamber (Scanvac, cool safe 90–80 A, Labogene, Denmark) at 0.1 hPa from −15–25 °C for 2 h. No significant change in the sample structure was observed from this procedure. The microstructure of the freeze dried samples were observed under a scanning electron microscope (SEM) (EVO® HD, Carl Zeiss, Germany) at an accelerating voltage of 10 kV and a working distance of 7–8.5 mm in secondary electron mode. Magnification of 1Kx.was applied for observation.

### 2.3 Creep test

The creep test was performed using a hold until time test by using a texture analyzer (HD Plus, Texture Analyzer, Stable Micro Systems, UK) with a 50-kg load cell. A 5 g sample of cooked rice was compressed up to 20 N maximum force using a deformation speed of 0.5 mm/s using the previously reported KMITL test rig (Sirisomboon et al., 2017). The force was kept constant, hence the stress was constant as the compression area was approximately constant. The KMITL test rig consists of two aluminum parts including the cylindrical compression probe with 30 mm in diameter and the cylindrical hole with 35 mm in diameter and 20 mm in height. The creep test was then performed. In the creep test, the maximum force was applied for 120 s, and the increase in deformation was recorded with time using Exponent version 6,1,5,0 (Stable Micro Systems, UK). The pre and post test speed was 5 and 10 mm/s, respectively. A typical graph representing the deformation over time was obtained.

### 2.4 Non-linear viscoelastic modeling using creep test data

Peleg (1980) explained the viscoelastic property with linear Eq. (1).

(1) $$\frac{\text{t}}{{\text{Y}}_{\text{t}}}={\;\text{k}}_{1}+{\;\text{k}}_{2}\text{t}\;\;\;\;\;$$

where t was time. ${\text{Y}}_{\text{t}}$ is a creep parameter which corresponds to the deformation, normalized deformation, strain $\left({\text{ε}}_{\text{t}}\right)$ or creep compliance. In this case the strain was used. ${\text{k}}_{1}$ and ${\text{k}}_{2}$ are constants which do not depended on retardation time. ${\text{k}}_{1}$ is the degree of solidity where $\frac{1}{{\text{k}}_{2}}$ is the asymptotic strain.

Purkayastha et al. (1984) proposed the creep model for biological material and food as follows:

(2) $${\text{Y}}_{\left(\text{t}\right)}\;={\;\text{k}}_{0}+{\;\text{k}}_{1}\text{t}+\frac{\text{t}}{{\text{k}}_{2}\;+{\;\text{k}}_{3}\text{t}}$$

${\text{k}}_{0}$ is a constant representing the instantaneous compliance ${\text{Y}}_{\left(\text{t}\right)}$, ${\text{k}}_{1}$ a constant representing the steady-state flow and ${\text{k}}_{2}$ and ${\text{k}}_{3}$ the characteristic constants of the creep function ${\text{Y}}_{\left(\text{t}\right)}$ obtained by regression from the linear relationship of $\left(\frac{\text{t}}{{\text{Y}}_{\left(\text{t}\right)}}\right)$ vs t.

In the proposed creep model below, when stress is constant and the initial strain is ${\text{ε}}_{0}$, the following equation is obtained:

(3) $${\text{ε}}_{\text{t}}\;={\;\text{ε}}_{0}\left(1+\;\frac{{\text{t}}^{\text{n}}}{{\text{k}}_{1}\;+{\;\text{k}}_{2}\;{\text{t}}^{\text{n}}}\right)$$

In this case, ${\text{Y}}_{\left(\text{t}\right)}$ is the creep compliance ($\frac{{\text{ε}}_{\text{t}}}{{\text{σ}}_{0}}$), ${\text{σ}}_{0}$ is the constant stress and, ${\text{E}}_{0}$ is the initial modulus of elasticity. The resultant equation can be described as:

(4) $$\frac{{\text{ε}}_{\text{t}}}{{\text{σ}}_{0}}\;=\;\frac{1}{{\text{E}}_{0}}\left(1+\;\frac{{\text{t}}^{\text{n}}}{{\text{k}}_{1}\;+{\;\text{k}}_{2}\;{\text{t}}^{\text{n}}}\right)$$

The non-linear viscoelastic modeling was conducted using a Gauss-Newton (GN) algorithm in the statistical package R (R Core Team, 2015). The constants of the optimum models from Eqs. (1), (2), and (3) were refined using the minimum residual standard error (RSE). The coefficient of determination (R^2^) was then calculated. The mean of nine replicates and the associated standard deviation for each constant was calculated. The significance of difference between the means was determined using a Duncan's multiple range test (DMRT) (Duncan, 1955) at a confidence level of 95% (p < 0.05).

### 2.5 Gauss-Newton (GN) algorithm

The GN algorithm can be used to solve a non-linear least square problem. It can be expressed mathematically as: $S=\overset{n}{\underset{j=1}{\sum }}r_{j}^{2}$, where S is the sum of the residuals, *r*_*j*_ is residual difference between the actual observed value (*y*_*j*_) and the predicted value by the model, $r_{j}=y_{j}-f\left(x_{j},\beta \right)$, *x* is the input value, *f* is the mathematical function trying to fit the data point, and $\beta =\left(\beta_{1}..\beta_{n}\right)$ is the parameters of the model aim at the minimization of *S* (Ho, 2018). The GN technique models three steps to estimate the parameters: 1) determining Jacobian, Z_j_; 2) determining residual vector, r_j_, and 3) evaluating solution matrix, *λ*_*(j+1)*_ (Hanief and Wani, 2015). For example, given the equation *f=a(1−e*^*-bx*^*)*, if the total data contains n points. Then, function has 2 parameters i.e. a and b. The dimension of Z is n×2 and the residual matrix (r) is n×1, and Z^T^Z is a 2×2 matrix. The Jacobian matrix is formed.

(5) $$Z_{j}={\left[\begin{array}{cc} \frac{\partial f}{\partial a_{1}} & \frac{\partial f}{\partial b_{1}}\\ \frac{\partial f}{\partial a_{2}} & \frac{\partial f}{\partial b_{2}}\\ \begin{array}{c} \vdots \\ \frac{\partial f}{\partial a_{n}} \end{array} & \begin{array}{c} \vdots \\ \frac{\partial f}{\partial b_{n}} \end{array} \end{array}\right]}_{n\times 2}$$

The equation for the GN algorithm is: (Z^T^Z)λ = –Z^T^r, where λ is the adjustment parameters. It is solved as: λ = −(Z^T^Z)^−1^Z^T^r, and λ_(j+1)_ = λ_(j−1)_+λ.

(6) $${\text{λ}}_{\text{(j}+1\text{)}}=\left[\begin{array}{c} a\\ b \end{array}\right]$$

Parameters a and b are initially selected arbitrarily to evaluate λ_1_. The effective values of λ_j+1_ were obtained by successive iterations using λ_(j+1)_ = (Z^T^Z)^−1^Z^T^r (Hanief and Wani, 2015).

## 3 Results and discussions

**Fig. 1:**
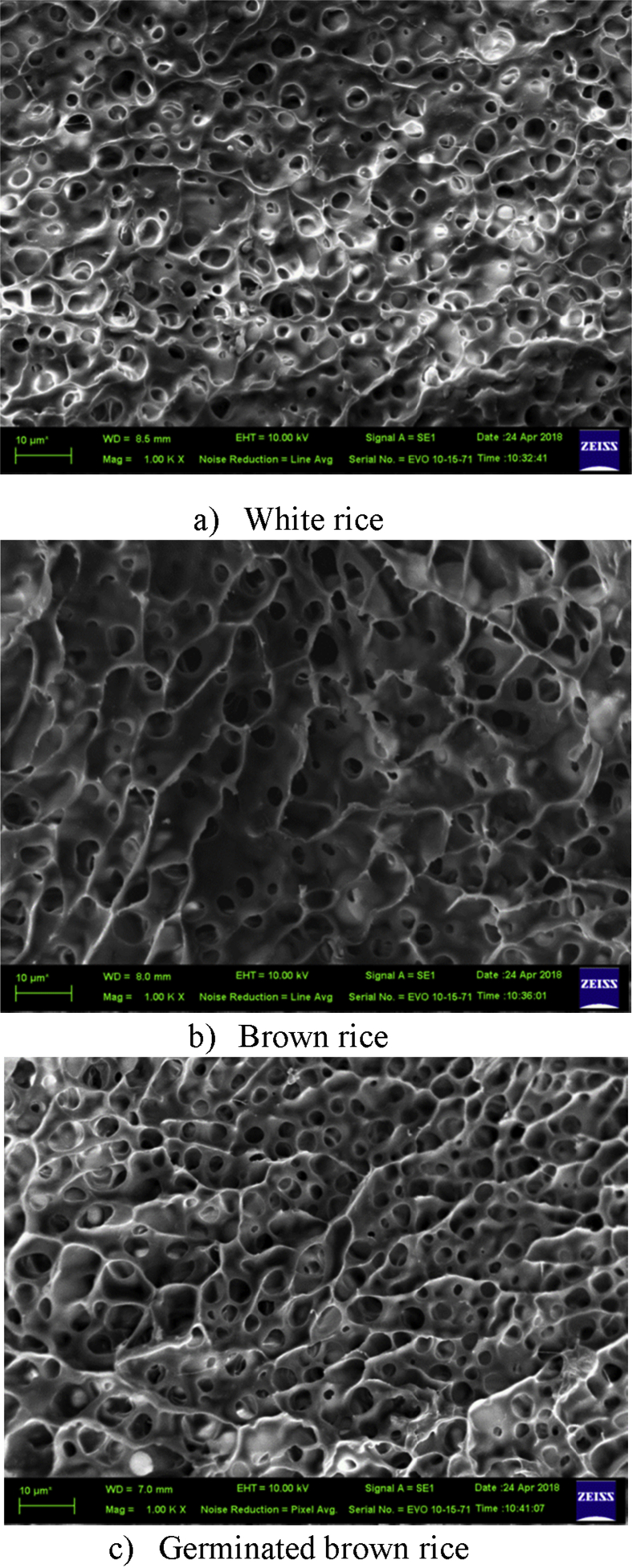
Microstructure of dried cooked rice observed by scanning electron microscopy (SEM).

The moisture content of the white rice, brown rice and germinated brown Thai Jasmine rice samples were observed to be 54.69 ± 0.97, 70.68 ± 1.51 and 56.86 ± 1.43 %wb, respectively. Fig. 1a–c reveal the morphology of the surface of the frozen dried cooked white, brown, and germinated brown rice, respectively from SEM analysis. All types of cooked rice had porous structure. The white rice had uniform smaller pore size compared to those of brown rice and germinated brown rice. The pore size of brown rice was largest. This might be because the highest water to rice ratio while cooking. The germinated brown rice had different pore size distributed in layers.

**Fig. 2:**
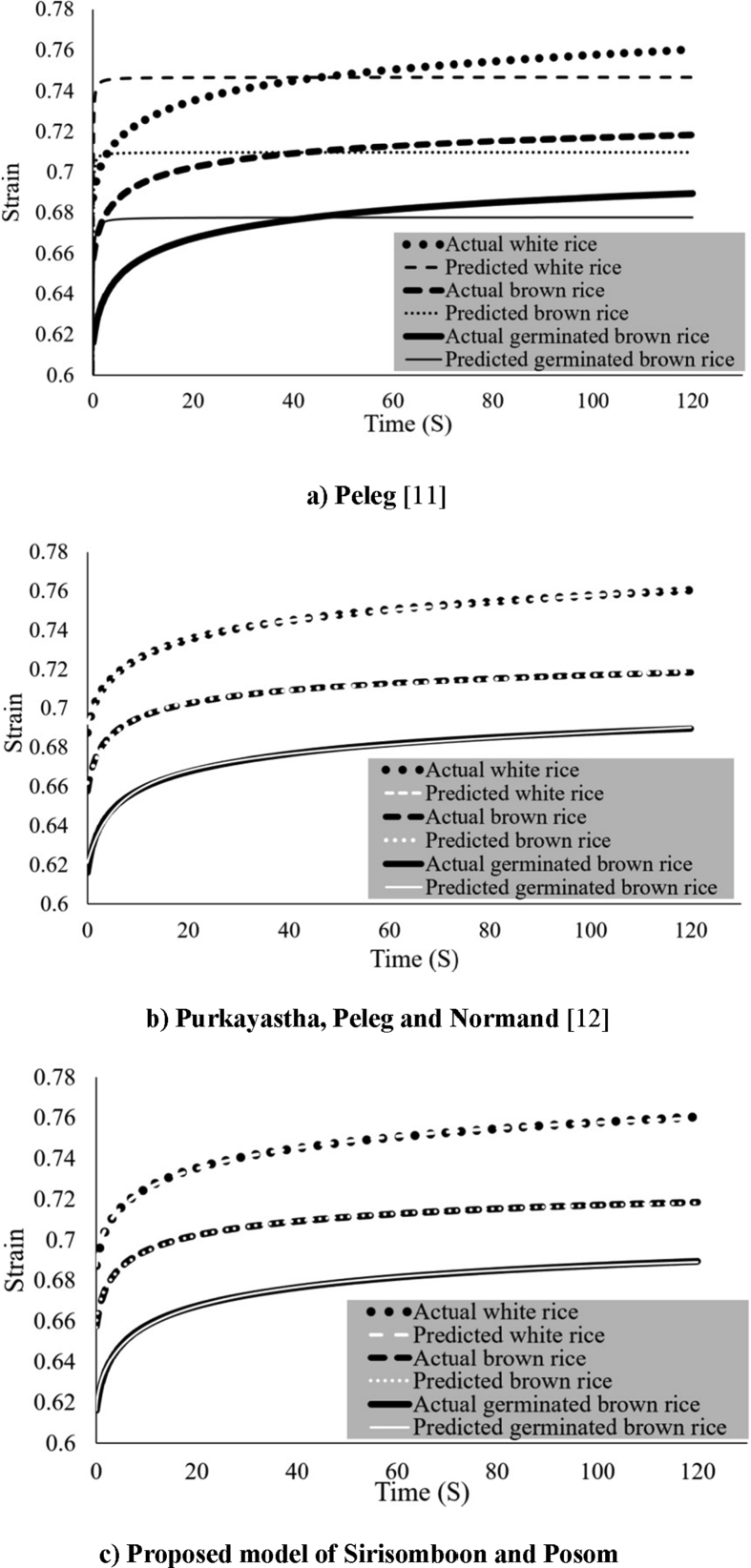
A typical measurement data and creep curves fitted for cooked rice by different models.

Fig. 2a shows the corresponding creep curves fitted using the Peleg (1980) model. Fig. 2b shows the curves fitted using the Purkayastha et al. (1984) model and Fig. 2c shows those fitted using the newly proposed model by Sirisomboon and Posom. Table 1 contains the viscoelastic parameters obtained from creep test of the three different rice models. The model proposed here by Sirisomboon and Posom was the most accurate, displaying the highest coefficient of determination (R^2^), 0.9991–0.9992, and the lowest residual standard error (RSE), 0.00030–0.00040.

**Table 1:** Creep parameters of non-linear viscoelastic models for cooked Thai jasmine rice samples.

Cooked Thai Jasmine rice samples		k_0_	k_1_	k_2_	k_3_	n	R^2^	RSE
White rice	Eq. (1)		0.010 ± 0.003a	1.43 ± 0.06b			0.0455	0.01292
	Eq. (2)	0.65 ± 0.03a	0.0001 ± 0.0000a	137.8 ± 31.4a	17.21 ± 1.59a		0.9989	0.00043
	Eq. (3)		41.88 ± 12.52a	6.24 ± 0.68a		0.59 ± 0.04b	0.9991	0.0004
Brown rice	Eq. (1)		0.009 ± 0.004a	1.44 ± 0.05b			0.054	-
	Eq. (2)	0.64 ± 0.03a	0.0001 ± 0.0000b	107.9 ± 22.8a	17.72 ± 1.73a		0.9982	0.00042
	Eq. (3)		35.00 ± 8.30a	7.07 ± 1.21a		0.60 ± 0.05b	0.9991	0.0003
Germinated brown rice	Eq. (1)		0.013 ± 0.008a	1.50 ± 0.04a			0.0592	0.01218
	Eq. (2)	0.61 ± 0.03b	0.0001 ± 0.0000b	116.1 ± 62.1a	17.07 ± 2.73a		0.9983	0.00052
	Eq. (3)		41.60 ± 16.14a	6.76 ± 1.09a		0.65 ± 0.02a	0.9992	0.00037

Eq. (1): $\frac{\text{t}}{{\text{ε}}_{\text{t}}}={\;\text{k}}_{1}+{\;\text{k}}_{2}\text{t}$, Eq. (2): ${\;\text{ε}}_{\text{t}}={\;\text{k}}_{0}+{\;\text{k}}_{1}\text{t}+\frac{\text{t}}{{\text{k}}_{2}\;+{\;\text{k}}_{3}\text{t}}$, and Eq. (3) (proposed model): ${\text{ε}}_{\text{t}}={\;\text{ε}}_{0}\left(1+\;\frac{{\text{t}}^{\text{n}}}{{\text{k}}_{1}\;+{\;\text{k}}_{2}\;{\text{t}}^{\text{n}}}\right)\text{.}$ R^2^ is coefficient of determination. RSE is residual standard error. Mean values in a column of the same equation followed by different letters (a–c) differ significantly (*p* < 0.05).

The k_1_ parameter is a constant that influences the creep process at the beginning. 1/k_1_ value describes the initial increasing rate when t is small. A high k_1_ value indicates that the initial increasing rate is low, the slope of the strain and time curve is small and the strain slowly approaches a constant value which indicates that the material has high elastic behavior. The k_1_ value of cooked white rice, brown rice and germinated brown rice were not found to be significantly different, suggesting they display the same elastic behavior. The 1/k_2_ value indicates the level of hypothetical asymptotic of strain that corresponds to the equilibrium strain level. When the k_2_ value increases the asymptotic strain decreases. When the k_2_ value is equal to infinity the material displays ideal elasticity and there will be no creep. When the k_2_ value is equal to zero, the material is an ideal liquid. The observation that the k_1_ and k_2_ values of all three rice samples were not significantly different indicates that they have the same elastic behavior. The n value is a constant that controls the approaching rate to the equilibrium strain. The n value is always greater than zero. The higher the n value, the quicker the initial increasing rate of strain is reached. Therefore the slope of the strain-time curve is high. The n value has no affect on the value of the equilibrium strain. From Table 1, the n values associated with the cooked germinated brown rice samples are found to be significant higher than those of cooked white rice and brown rice. The n values of the cooked white rice and brown rice were not significantly different. This indicates that the germinated brown rice will creep faster at the beginning of the test, after it has been subjected to a constant stress. This was supported by the microstructure of germinated brown rice by SEM analysis that showed (Fig. 1c) the pore distribution in layers when compared to those of brown rice and white rice.

Generally the creep behavior of food follows linear creep behavior using the classical models, for examples on rice gel; from Xu et al. (2008), the creep process of rice gel consisted mainly of retarded elastic deformation, and viscosity flow deformation. The retarded elastic modulus, relaxation time, and the viscosity coefficient, of the rice gel were estimated according to the Burger model; and wheat kernel, the creep tests of wheat kernels were studied with the generalized Kelvin–Voigt model (Hernandez-Estrada et al., 2012).

## 4 Conclusion

The proposed creep model by Sirisomboon and Posom in this work showed good performance in predicting the non linear viscoelastic parameters from creep test for three different types of Thai Jasmine rice (milled rice, brown rice and germinated brown rice). The three rice variants displayed the same elastic behavior, however, the germinated brown rice was observed to creep faster at the beginning of the test, after it was subjected to constant stress. There are several advantages of evaluating non linear viscoelastic properties on cooked rice of different processes, including: 1) it is the basic final form of rice usage that customer consumes; 2) the viscoelastic properties obtained from modeling is fundamental rheological characterization that readily to correlated with the sensory properties of the cooked rice, 3) the non linear viscoelastic tests on cooked rice are easy to perform by the developed protocol. The application of non linear viscoelastic model can also be applied for food other than rice.

## Declarations

### Author contribution statement

Jetsada Posom, Panmanas Sirisomboon: Conceived and designed the experiments; Performed the experiments; Analyzed and interpreted the data; Contributed reagents, materials, analysis tools or data; Wrote the paper.

### Funding statement

This research did not receive any specific grant from funding agencies in the public, commercial, or not-for-profit sectors.

### Competing interest statement

The authors declare no conflict of interest.

### Additional information

No additional information is available for this paper.

## References

[bib1] Bockstaele F.V., Leyn I.D., Eeckhout M., Dewettinck K. (2011). Non-linear creep-recovery measurements as a tool for evaluating the viscoelastic properties of wheat flour dough. J. Food Eng..

[bib2] Cáceres P.J., Peñas E., Martine C. (2017). Enhancement of biologically active compounds in germinated brown rice and the effect of sun-drying. J. Cereal Sci..

[bib3] Cho D., Lim S. (2016). Germinated brown rice and its bio-functional compounds. Food Chem..

[bib4] R Core Team (2015). R: a Language and Environment for Statistical Computing. http://www.R-project.org.

[bib5] Duncan D.B. (1955). Multiple range and multiple F tests. Biometrics.

[bib6] Hanief M., Wani M.F. (2015). Modeling and prediction of surface roughness for running-in wear using Gauss-Newton algorithm and ANN. Appl. Surf. Sci..

[bib7] Hernandez-Estrada Z.J., Figueroa J.D.C., Rayas-Duarte P., Pena R.J. (2012). Viscoelastic characterization of glutenins in wheat kernels measured by creep tests. J. Food Eng..

[bib8] Ho N. (2018). Gauss Newton Algorithm. http://nghiaho.com/?page_id=355.

[bib9] Martinez A., Nieto A., Viollaz P., Alzamora S. (2005). Viscoelastic behavior of melon tissue as influenced by blanching and osmotic dehydration. J. Food Sci..

[bib10] Myhan R., Białobrzewski I., Markowski M. (2012). An approach to modeling the rheological properties of food materials. J. Food Eng..

[bib11] Peleg M. (1980). Linearization of relaxation and creep curves of solid biological materials. J. Rheol..

[bib12] Purkayastha S., Peleg M., Normand M. (1984). Presentation of the creep curves of solid biological materials by a simplified mathematical version of the generalized Kelvin-Voigt model. Rheol. Acta.

[bib13] Simonelli C., Galassi L., Cormegna M., Bianchi P. (2017). Chemical, physical, textural and sensory evaluation on Italian rice varieties. Univ. J. Agric. Res..

[bib14] Sirisomboon P., Kaewsorn K., Thanimkarn S., Phetpan K. (2017). Non-linear viscoelastic behavior of cooked white, brown and germinated brown Thai jasmine rice by large deformation relaxation test. Int. J. Food Prop..

[bib15] Sozer N., Kaya A., Dalgic A. (2008). The effect of resistant starch addition on viscoelastic properties of cooked spaghetti. J. Text. Stud..

[bib16] Truong V.D., Daubert C.R. (2000). Comparative study on large strain methods in assessing failure characteristics of selected food gels. J. Text. Stud..

[bib17] Xu Y.-L., Xiong S.-B., Li Y.-B., Zhao S.-M. (2008). Study on creep properties of indica rice gel. J. Food Eng..

[bib18] Zhang J., Christopher R., Daubert E., Foegeding A. (2007). A proposed strain-hardening mechanism for alginate gels. J. Food Eng..

